# Application of metal stent implantation with endoscope and X-ray fluoroscopy combined laparoscopic surgery in the treatment of acute left hemicolon cancer obstruction

**DOI:** 10.1186/s12957-023-03228-x

**Published:** 2023-10-21

**Authors:** Xiao-Cong Zhou, Fei-Yue Ke, Gaurav Dhamija, Ruchi D. Viroja, Chun-Wei Huang

**Affiliations:** 1 Department of Colorectal Surgery, The Dingli Clinical Institute of Wenzhou Medical University (Wenzhou Central Hospital), Wenzhou, Zhejiang People’s Republic of China; 2 Postgraduate Training Base Alliance of Wenzhou Medical University (Wenzhou Central Hospital), Wenzhou, Zhejiang People’s Republic of China; 3 School of International Studies, Wenzhou Medical University, Wenzhou, Zhejiang People’s Republic of China; 4 Department of Gastroenterology, The Dingli Clinical Institute of Wenzhou Medical University (Wenzhou Central Hospital), No.252, Baili East Road, 325000 Wenzhou, Zhejiang People’s Republic of China

**Keywords:** Endoscope, Laparoscopic surgery, Metal stent implantation, Cancerous obstruction

## Abstract

**Background:**

This study aimed to conduct a case–control study of endoscopic and fluoroscopic metal stent placement combined with laparoscopic surgery versus conventional open Hartmann’s procedure in treating acute left-sided colon cancer obstruction. Additionally, the study aims to discuss the application value of endoscopic and X-ray-guided metal stent placement combined with laparoscopic surgery in the treatment of acute left-sided colon cancer obstruction.

**Methods:**

From June 2011 to December 2019, 23 patients with acute left-sided colon cancer obstruction who underwent metal stent implantation combined with laparoscopic surgery under endoscopy and X-ray fluoroscopy in Wenzhou Central Hospital were collected, and 20 patients with acute left-sided colon cancer obstruction who underwent traditional emergency open Hartmann’s surgery during the same period were selected as a control group. All patients were diagnosed with left colon obstruction by plain abdominal film and/or CT before the operation and colon adenocarcinoma by colonoscopic biopsy and/or postoperative pathology. The operation time, intraoperative blood loss, postoperative anal exhaust time, the success rate of one-stage anastomosis, postoperative hospital stay, and postoperative complications were compared between the two groups.

**Results:**

This study showed a significant difference in the therapeutic effect between the two groups. Compared with the traditional Hartmann’s operation group, the success rate of one-stage anastomosis in endoscopic and X-ray-guided metal stent placement combined with the laparoscopic operation group was significantly higher than that in the Hartmann’s operation group (*P* < 0.05). The overall incidence of postoperative complications and hospital stay were significantly lower in the observation group than in the Hartmann’s group (*P* < 0.05). Further subgroup analysis of the overall postoperative complication rate of the two groups showed that the traditional Hartmann’s operation group was more likely to have an incomplete intestinal obstruction (*P* < 0.05). This study also showed no significant differences between the two groups in operation time, intraoperative blood loss, number of harvested lymph nodes, and postoperative anal exhaust time (all *P* > 0.05). This study also found no significant differences between the two groups in overall survival rates or recurrence-free survival rates (all *P* > 0.05).

**Conclusions:**

The comparison of the therapeutic effects of the two groups verified the feasibility of endoscopy combined with X-ray fluoroscopy metal stent placement in combination with laparoscopic surgery in the treatment of acute left-sided colon cancer obstruction. Compared with the traditional emergency open Hartmann’s procedure, metal stent implantation under endoscopy and X-ray fluoroscopy combined with laparoscopic surgery is more minimally invasive, safe, and effective. It avoids the traditional second or even third surgical trauma to effectively improve the quality of life of patients, so that patients can recover quickly after surgery.

## Introduction

Colorectal cancer is one of the most common malignant tumors. The incidence and mortality of colorectal cancer are increasing in China [1]. Colorectal cancer complicated with acute intestinal obstruction is one of the common acute abdominal diseases in surgery. About 7–29% of colorectal cancer patients present with acute complete or incomplete intestinal obstruction symptoms [2, 3]. Approximately 70% of colorectal malignant obstruction is in the left half of the colon [4]. The scope of the left colon generally includes the left half of the transverse colon, the splenic flexure of the colon, the descending colon, and the sigmoid colon, and the upper part of the rectum also belongs to the category of the left colon in a broad sense [5]. Complete obstruction of left hemicolon cancer is categorized as a closed-loop obstruction, which necessitates immediate treatment to alleviate the obstruction. If left untreated, this obstruction can lead to various severe complications such as water and electrolyte imbalance, acid–base disorders, intestinal wall ischemia, necrosis, and perforation. It may also result in conditions like acute diffuse peritonitis, septic shock, and other life-threatening complications. Additionally, it is worth noting that patients with proximal colon obstruction often exhibit dilation and edema due to the lack of routine preoperative bowel preparation in these cases. The diameter difference between proximal and distal intestinal tubes is significant, which is not conducive to anastomosis. In addition, anatomical and physiological factors such as a relatively thin wall of the left colon, poor blood supply of the colon, and many bacteria were found. Additionally, most patients were elderly, often complicated with various underlying diseases, long course of the disease, anemia, hypoproteinemia, and internal environmental disorders [4, 6]. There is a high risk of postoperative complications, especially anastomotic leakage [6]. Therefore, the safety of primary tumor resection and anastomosis is still controversial [4, 6, 7].

There is no unified standard for treating acute left-sided colon cancer obstruction. Traditional surgery is mainly based on open surgery, and staging surgery is primarily used ((1) The two-stage operation was Hartmann’s procedure, the first emergency operation of stage I tumor resection and relief of intestinal obstruction, proximal colostomy, distal closure, and then stage II colostomy closure. (2) Three-stage surgery, including stage I proximal colostomy to relieve intestinal obstruction, stage II tumor resection, and stage III closed stoma) or intraoperative irrigation with one-stage anastomosis. The former Hartmann’s procedure will cause the trauma of the second operation, prolong the patient’s recovery period, and increase the overall treatment cost. At the same time, the abdominal stoma also increases the psychological burden and pain of the patient, and the second operation also increases the difficulty and risk of the surgeon. However, the three-stage surgery, with the same surgical trauma and many complications, not only increases the pain and economic burden of patients but also increases the risk of tumor spread, making some patients lose the opportunity for radical surgery [4–6, 8].

Moreover, it is difficult or even impossible to close the stoma in 40–60% of patients after staged surgery, resulting in permanent stoma [9]. The latter is not recognized by most clinicians because intestinal lavage prolongs the operation time and increases the chance of abdominal cavity contamination. At the same time, due to intestinal wall edema and hypoproteinemia, postoperative complications such as anastomotic leakage, abdominal cavity, and wound infection are as high as about 30% [8, 10].

None has yet been accepted as the best treatment for left-sided colon cancer obstruction. The ideal treatment method is to solve the obstruction of the left colon without surgery, or at least surgery should be performed with proper bowel preparation and good general condition for selective surgical resection and essential anastomotic connection. The placement of metal stents under endoscopy combined with X-ray fluoroscopy has made it possible. In many cases of malignant digestive tract obstruction, this method has become a partial alternative to surgery.

## Materials and methods

### Patients

The case data of 23 patients with acute left hemi-colon cancerous obstruction treated by endoscopic combined with X-ray fluoroscopic metal stent placement combined with laparoscopic surgery in Wenzhou Central Hospital were collected between June 2011 and December 2019 (observation group), of whom 15 were male, and 8 were female. Twenty patients with acute left hemi-colon cancerous obstruction who underwent Hartmann’s procedure as a traditional emergency open surgery simultaneously were used as controls (control group), of whom 9 were male and 11 were female.

(A) Inclusion criteria: ① enrolled cases with complete clinical and pathological data, all with preoperative confirmation of left hemicolectomy obstruction by plain abdominal radiograph and/or CT and a diagnosis of adenocarcinoma of the left hemicolectomy by colonoscopic biopsy and/or postoperative pathology; ② adult patients aged 18 years and over with left colon cancer complicated with intestinal obstruction; ③ abdominal plain radiograph and/or CT showing that the lower border of the tumor obstructing the intestinal lumen lies between the splenic flexure of the colon and the sigmoid junction of the rectum; and ④ these procedures are performed by skilled interventional gastrointestinal endoscopists and colorectal surgeons. General anesthesia is used during all colorectal surgery.
(B) Exclusion criteria: ① general condition or other comorbidities that do not tolerate general anesthesia and are unsuitable for surgical treatment; ② pre-operative assessment of the tumor for non-radical resection; ③ previous history of major abdominal surgery; ④ strangulated intestinal obstruction or peritonitis; and ⑤ physical examination revealed a large mass palpable in the whole abdomen. It is suspected that heavy abdominal adhesions or large tumor size may easily lead to conversion to open surgery during laparoscopic colorectal resection.
(C) Selection of clinicopathological parameters: the patient’s age, gender, body mass index (BMI), degree of intestinal obstruction, location of intestinal obstruction, electrolyte disturbance, anemia, maximum diameter of tumor, depth of tumor invasion (T stage), lymph node metastasis (*N* stage), tumor stage [TNM (tumor–node–metastasis) stage], degree of tumor differentiation, vascular tumor thrombus, nerve invasion, cancer nodule, operation time, intraoperative blood loss, postoperative anal exhaust time, postoperative hospitalization time, and postoperative complications, where BMI = weight/height squared (international unit kg/m^2^). Tumor staging was based on the TNM staging system of AJCC 8th edition [11]. Calculation of operative time: laparoscopic surgery from the beginning of the trocar placement into the abdominal cavity to the end of the suturing to the abdomen, Hartmann’s procedure from the beginning of the skin incision to the end of the suturing to the abdomen; calculation of intraoperative bleeding: the total amount of blood attracted by the suction device collected in the suction bottle and the use of gauze were converted before abdominal closure and pelvic irrigation.

### Methods and surgical procedures

1. Metal stent placement combined with laparoscopic surgery group: after definite obstruction, preoperative gastrointestinal decompression, and metal stent placement in the colon under colonoscopy combined with X-ray fluoroscopy: After the tumor was observed under transanal colonoscopy, a guide wire was placed through the stenosis under X-ray fluoroscopy, the guide wire was introduced into the catheter, and the contrast agent was injected through the catheter. The condition of the distal digestive tract and the length of the stenosis were observed by angiography. The appropriate stent model was selected according to the stenosis length, ensuring that both ends of the stent exceeded the lesion by more than 2 cm. After the transcatheter injection of the contrast agent, the guide wire was inserted again. After the guide wire was inserted, the tumor was biopsied for pathological examination. Finally, the stent was placed along the guide wire under the dual observation of endoscopy and X-ray, and the principle of “placing while pulling” was followed. That is, the ostial end was satisfied first, and after the ostial end was observed to be opened under X-ray fluoroscopy, the stent was pulled to the anal end while being released. The proximal end was accurately positioned before the stent was completely released. If the proximal position was insufficient after placement, foreign body forceps could pull the stent proximally under X-ray fluoroscopy. The treatment of sigmoid carcinoma with intestinal obstruction by metal stent implantation under an endoscope combined with X-ray fluoroscopy is shown in Fig. 1.

**Fig. 1:**
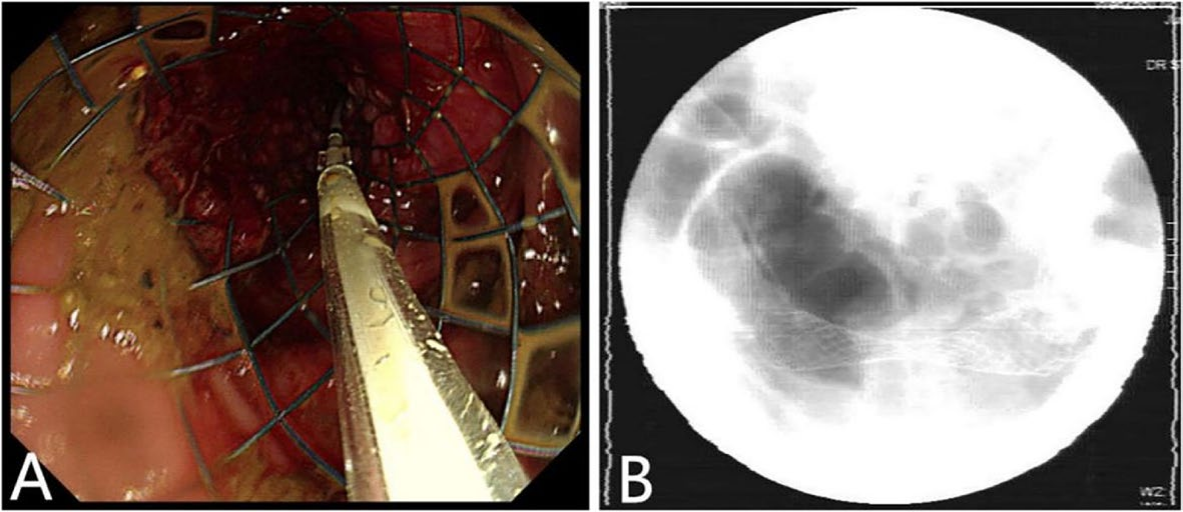
Endoscopic and fluoroscopic metal stent placement for sigmoid colon cancer with intestinal obstruction: **A** colonic stent was placed and released under the guidance of guidewire; **B** is the postoperative X-ray to understand the position of the stent
2. After the successful placement of the stent, the patient experienced a gradual improvement in symptoms such as abdominal pain and distension. Additionally, the patient began to experience relief from exhaustion and was able to defecate normally. To further enhance the patient’s preoperative condition, routine bowel preparation was conducted for a period of 7 to 14 days. Following this, laparoscopic radical resection was performed to treat the left hemicolon cancer. The laparoscopic left hemicolectomy procedure can be categorized into three types: laparoscopic descending colon cancer radical resection, laparoscopic sigmoid colon cancer radical resection, and laparoscopic rectosigmoid junction cancer radical resection. For primary intestinal anastomosis in cases of sigmoid colon cancer and rectosigmoid junction cancer, the double stapler technique was utilized. On the other hand, tumors located in the splenic flexure or descending colon underwent end-to-side anastomosis.
3. Traditional emergency Hartmann’s operation group: After the definite diagnosis of tumor intestinal obstruction, patients underwent adequate preoperative gastrointestinal decompression, emergency radical resection of left colon cancer + proximal colostomy + distal block, and strictly followed the principle of radical tumor resection during the operation. Subsequently, a colostomy was performed.

### Statistical analysis

The experimental data were analyzed by SPSS 20.0 statistical software or R software (version 4.2.1). The measurement data conforming to normal distribution were expressed as mean ± standard deviation (‾x ± s), and the measurement data not conforming to normal distribution were expressed as median (interquartile range). To compare the two groups, either the independent sample *t* test or rank sum test was used. The statistical data were represented by the number of cases (%), and the comparison between the two groups was conducted by the chi-square test or Fisher’s exact probability method. The Kaplan–Meier method was used to generate overall survival (OS) and recurrence-free survival (RFS), and the log-rank test was used to assess differences between groups. The test level was 0.05, and *P* < 0.05 was considered statistically significant.

## Results

### Data analysis

The general data and clinicopathological stage of the two groups are shown in Table 1. The study found that there was a difference in electrolyte disturbance between the two groups (*P* < 0.05). However, there were no significant differences in the baseline data for other factors between the two groups (all *P* > 0.05). This suggests that, apart from electrolyte disturbance, the data for the two groups were balanced and comparable. (The postoperative pathology of the two groups showed that the upper and lower resection margins and circumferential resection margins were negative).

**Table 1:** Comparison of baseline information of patients in the observation and control groups

	Observation group	Control group	Value of statistics	*P*
	*n* = 23	*n* = 20	*t/z*或*χ*^2^	
**Gender [*n* (%)]**			*χ*^2^ = 1.773	0.183
Male	15 (65.2)	9 (45.0)		
Female	8 (34.8)	11 (55.0)		
**Age(years)**	66.7 ± 10.5	67.8 ± 11.2	*t* = 0.320	0.751
**BMI (kg/m**^**2**^**)**	22.65 ± 2.95	21.69 ± 4.41	*t* = 0.851	0.400
**Degree of obstruction [*n* (%)]**			*χ*^2^ = 0.183	0.669
Complete	13 (56.5)	10 (50.0)		
Incomplete	10 (43.5)	10 (50.0)		
**Site of obstruction [*n* (%)]**			*χ*^2^ = 3.478	0.349
Flexure of spleen	2 (8.7)	2 (10.0)		
Descending colon	10 (43.5)	4 (20.0)		
Sigmoid colon	8 (34.8)	12 (60.0)		
Straight B junction	3 (13.0)	2 (10.0)		
Maximum diameter of tumor (cm)	4.5 (3.5,5.1)	4.75 (4.0, 5.0)	*z* = 0.087	0.931
**Anemia [*n* (%)]**			*χ*^2^ = 1.713	0.191
(+)	7 (30.4)	10 (50.0)		
(−)	16 (69.6)	10 (50.0)		
**Electrolyte disturbances [*n* (%)]**			*χ*^2^ = 5.969	**0.015**
(+)	13 (56.5)	4 (20.0)		
(−)	10 (43.5)	16 (80.0)		
**Pathological T staging [*n* (%)]**			*χ*^2^ = 2.618	0.106
T1	0	0		
T2	0	0		
T3	17 (73.9)	10 (50.0)		
T4	6 (26.1)	10 (50.0)		
**Pathological *N* staging [*n* (%)]**			*χ*^2^ = 4.923	0.162
N0	9 (39.1)	4 (20.0)		
N1	12 (52.2)	9 (45.0)		
N2	2 (8.7)	6 (30.0)		
N3	0	1 (5.0)		
**Pathology TNM staging [*n* (%)]**			*χ*^2^ = 2.199	0.138
I	0	0		
II	8 (34.8)	3 (15.0)		
III	15 (65.2)	17 (85.0)		
**Degree of tumor differentiation [*n* (%)]**			*χ*^2^ = 2.453	0.886
Low differentiation	1 (4.3)	0		
Medium–low differentiation	7 (30.4)	5 (25.0)		
Intermediate differentiation	13 (56.5)	13 (65.0)		
High school differentiation	1 (4.3)	2 (10.0)		
High degree of differentiation	1 (4.3)	0		
**Tumor emboli in blood vessels [*n* (%)]**			*χ*^2^ = 1.992	0.158
(+)	13 (56.5)	7 (35.0)		
(−)	10 (43.5)	13 (65.0)		
**Neurocarcinoma invasion [*n* (%)]**			*χ*^2^ = 0.467	0.494
(+)	8 (34.8)	9 (45.0)		
(−)	15 (65.2)	11 (55.0)		
**Cancer nodule [*n* (%)]**			*χ*^2^ = 0.048	0.826
(+)	4 (17.4)	4 (20.0)		
(−)	19 (82.6)	16 (80.0)		

### Comparison of treatment effects between the two groups

The comparison of treatment effects between the trial and control groups is summarized in Table 2. The success rate of one-stage anastomosis in the endoscopic and X-ray-guided metal stent implantation combined with the laparoscopic surgery group (observation group) was significantly higher than that in the Hartmann’s procedure group (control group). The overall incidence of postoperative complications and postoperative hospital stay in the Hartmann’s operation group were significantly lower than those in the control group, and the differences were statistically significant (*P* < 0.05). There were no significant differences between the two groups in operation time, intraoperative blood loss, number of harvested lymph nodes, and postoperative anal exhaust time (all *P* > 0.05).

**Table 2:** Comparison of treatment effects between the observation and control groups

	Observation group (*n* = 23)	Control group (*n* = 20)	Value of statistics *t/z*或*χ*^2^	*P*
Operation time (min)	230.0 (205.0, 280.0)	202.5 (173.8, 315.0)	*z* = 0.939	0.348
Blood loss (ml)	50.0 (50.0, 100.0)	50.0 (50.0, 100.0)	*z* = 0.527	0.598
Postoperative anal exhaust time (d)	3.0 (3.0, 4.0)	2.5 (2.0, 3.8)	z = 1.221	0.222
Postoperative hospital stay (d)	12.2 ± 4.4	19.3 ± 5.8	*t* = 4.577	**0.000**
The number of lymph node dissections [PCS]	18.5 ± 5.9	15.6 ± 7.8	*t* = 1.395	0.171
Overall postoperative complications^a^[*n* (%)]	2 (8.7)	8 (40)	χ^2^ = 5.874	**0.015**
Postoperative wound infection	1 (4.3)	1 (5.0)		1.000
Subcutaneous fluid collection	0	1 (5.0)		0.465
Postoperative intra-abdominal infection	1 (4.3)	1 (5.0)		1.000
Pulmonary infection	1 (4.3)	1 (5.0)		1.000
Postoperative incomplete intestinal obstruction	0	4 (20.0)		**0.039**
Postoperative circulatory complications	0	1 (5.0)		0.465

^a^One patient in the observation group had postoperative incision infection and pulmonary infection. One patient in the control group had postoperative incision infection and incomplete intestinal obstruction

### Survival analysis and long-term results

The study included a total of 23 cases in the observation group and 20 cases in the control group. Through follow-up data analysis, 5 cases were lost to follow-up, with 1 case from the observation group and 4 cases from the control group. There were 24 deaths in total, comprising 13 deaths in the observation group, and 11 in the control group. Across both study groups, there were 22 total cases of tumor recurrence, including 13 recurrences in the observation group and 9 recurrences in the control group.

**Fig. 2:**
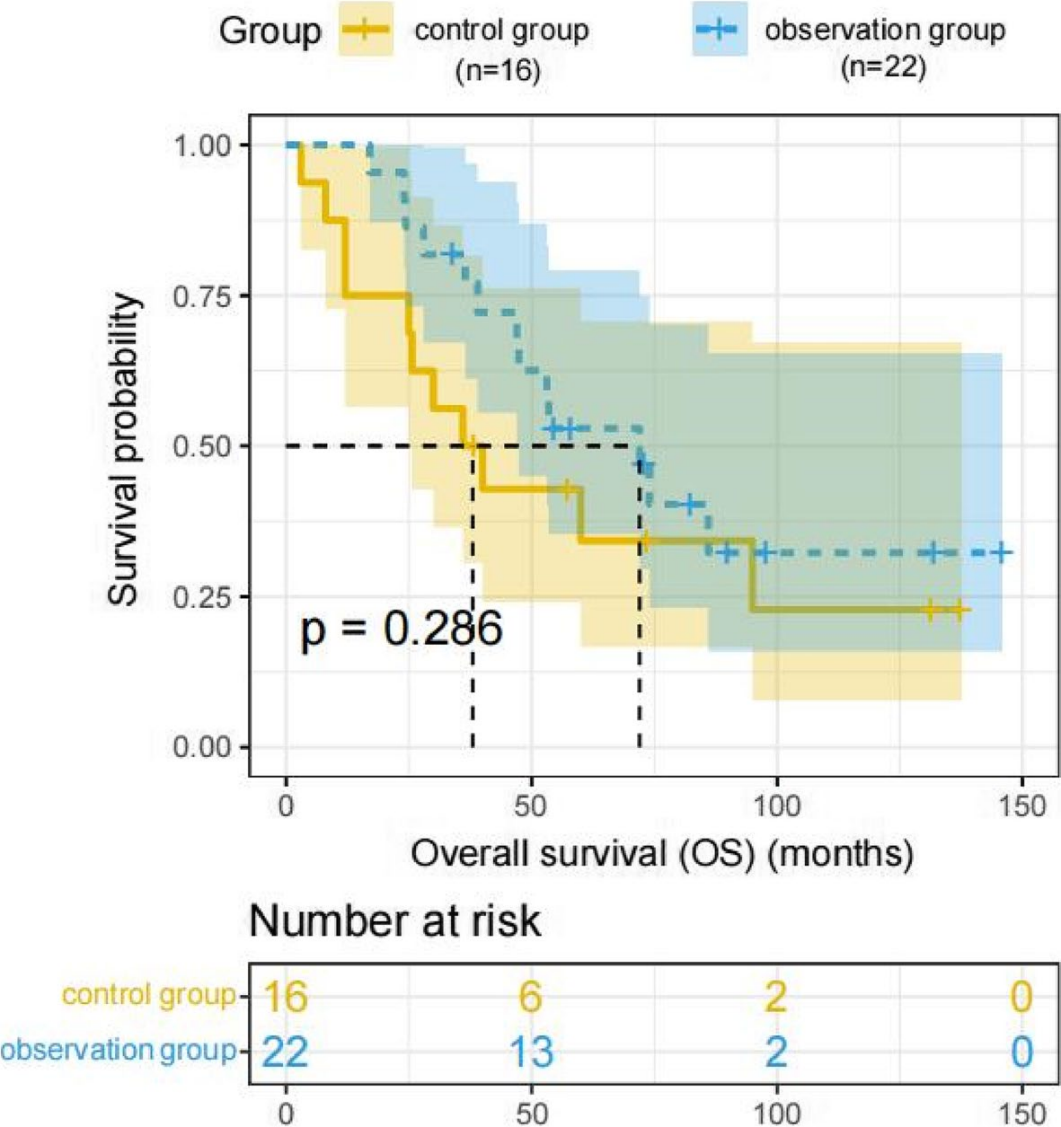
Comparison of overall survival (OS) between the observation and control group (*P* value 0.286). *P* values are from the log-rank test

**Fig. 3:**
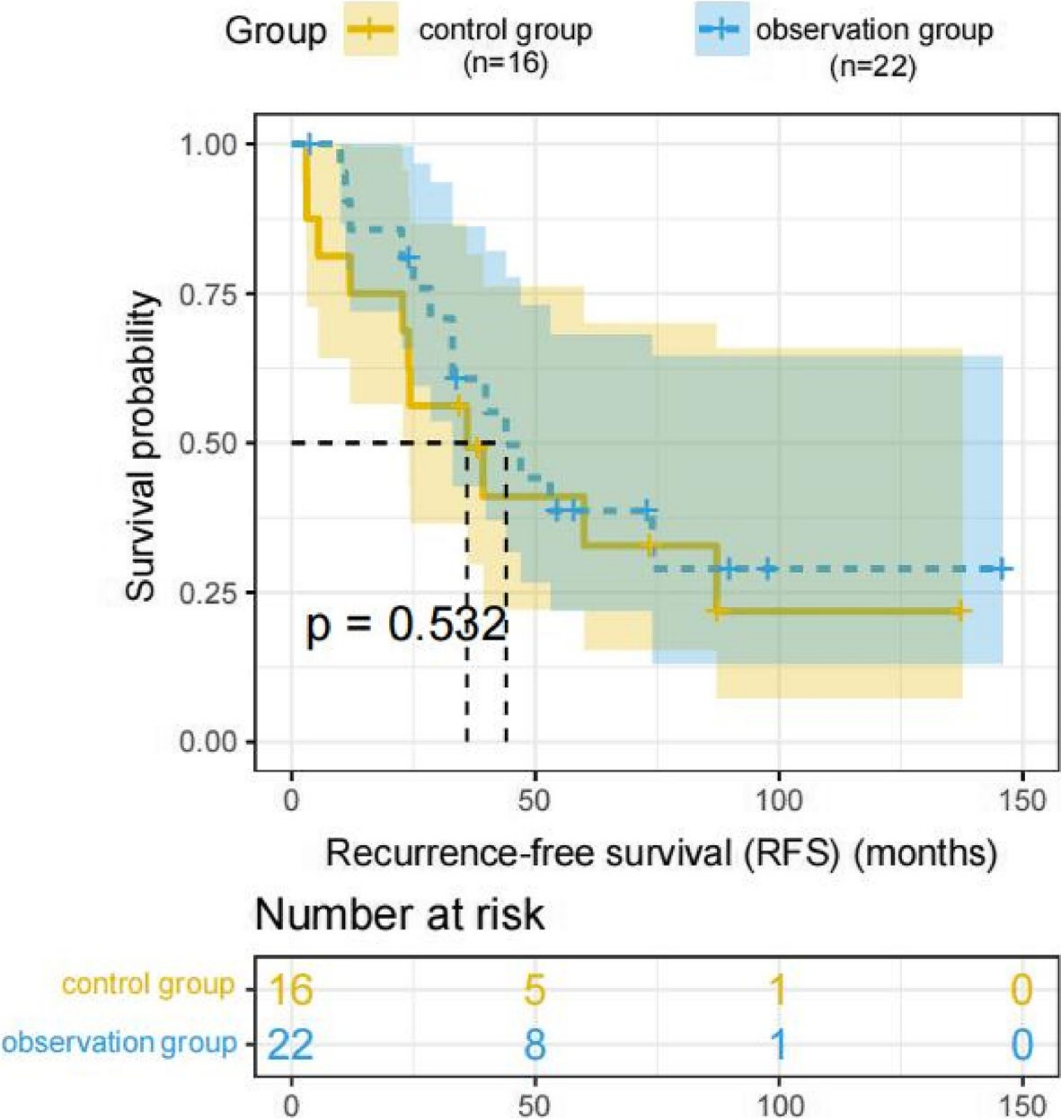
Comparison of recurrence-free survival (RFS) between the observation and control group (*P* value 0.532). *P* values are from the log-rank test

Kaplan–Meier analysis showed that the median overall survival time was 72 months in the observation group, compared to 36 months in the control group. The median recurrence-free survival time was 44 months in the observation group versus 36 months in the control group. Log-rank testing revealed no statistically significant differences between the two groups in the OS rates and RFS rates (*P* values were 0.286 and 0.532, respectively) (Figs. 2 and 3).

## Discussion

Colorectal cancer is currently one of the most common malignancies in the world, and current data show that the incidence of colorectal cancer has become the third most common malignancy worldwide [12]. In China, the incidence of colorectal cancer is the fourth highest among women and the fifth highest among men [13]. In our country, patients with colon cancer are often in the progressive stage at the time of diagnosis, and some patients with left-sided colon cancer present with acute bowel obstruction as the primary presentation [14]. Since complete obstruction of left hemicolon cancer belongs to closed-loop intestinal obstruction, emergency treatment is needed to relieve the obstruction. If left untreated, this obstruction can lead to various severe complications such as water and electrolyte imbalance, acid–base disorders, intestinal wall ischemia, necrosis, and perforation. It may also result in conditions like acute diffuse peritonitis, septic shock, and other serious complications.

For treating acute right hemicolectomy with intestinal obstruction, there is a consensus that a stage I right hemicolectomy + ileocolonic anastomosis should be performed if the tumor is resectable [15]. However, whether to perform one-stage tumor resection and anastomosis for acute left-sided colon cancer obstruction is still controversial [4, 6, 7]. Because there is no preoperative bowel preparation in such patients, the proximal colon of obstruction is usually dilated and edematous, the diameter of the distal and proximal bowel is quite different, and the intestinal wall of the left colon is relatively thin, the blood supply of the bowel is poor, and the amount of bacteria is large. In addition, most patients are elderly, often with various underlying diseases, a long course of disease, and many adverse factors such as low nutritional status and internal environmental disorders [4, 6]. In addition, intraoperative intestinal lavage is required before one-stage anastomosis, which prolongs the operation time and increases the chance of abdominal contamination [8, 10]. There is often a high risk of postoperative complications, especially anastomotic leakage [6]. It has been reported that the mortality rate of such emergency surgery is 15–20%, and the incidence of complications is as high as 40–50% [16].

Therefore, most scholars still advocate staging surgery [17]. Traditional staged surgery is mainly based on open surgery, and the surgical method is mostly used in two-stage surgery. Often Hartmann’s procedure, that is, emergency surgery, is performed. First, the tumor is removed, and the intestinal obstruction is relieved. The proximal colostomy is performed, the distal closure is performed, and the colostomy is withdrawn in the second stage. However, the Hartmann’s procedure will cause the trauma of the second operation for patients, prolong the recovery period of patients, and increase the overall treatment cost. At the same time, abdominal stoma also increases patients’ psychological burden and pain. For surgeons, the second operation also increases the difficulty and risk of the operation. However, stage III surgery, namely stage I proximal colostomy to relieve intestinal obstruction, stage II tumor resection, and stage III closed colostomy, has the same surgical trauma and many complications, which not only increases the pain and economic burden of patients but also increases the risk of tumor spread. Some patients have tumor metastasis and dissemination during reoperation and lose the opportunity for radical surgery [4–6, 8]. Therefore, three-stage surgery has been gradually abandoned. Moreover, it is difficult or even impossible to close the stoma in 40–60% of patients after staged surgery, resulting in permanent stoma [9].

Endoscopic placement of metal stents for malignant colorectal obstruction is a further development following the application of metal stents for malignant diseases of the esophagus, cardiac, and biliary tract. In 1991, Dohmoto et al. [18] first reported colon stent and applied it to treat acute left hemicolon obstruction caused by colonic tumors. Subsequently, many research groups in Europe, America, Japan, and other countries affirmed the application value of colon stents in treating colon cancer complicated with intestinal obstruction [19–21]. In the past 20 years, this treatment method has been promoted and improved worldwide. The clinical application of metal stent implantation under endoscopy combined with X-ray fluoroscopy can effectively relieve acute intestinal obstruction so that the surgeon can obtain sufficient preoperative preparation time, correct the patient’s nutritional status and water and electrolyte balance disorders, reduce intestinal inflammation and edema, and then perform the one-stage anastomosis. It acts as a “bridge” for one-stage anastomosis and turns emergency surgery into a limited operation. As a result, the incidence of stoma and infection and the burden of secondary closure of stoma were significantly reduced [4, 22, 23]. At present, the technology of colorectal stent implantation is becoming more and more mature. It is reported that the technical and clinical success rates of colorectal stent implantation are 90–100% and 84–94%, respectively [6, 24, 25]. In this study, the technical and clinical success rates of 23 experimental group patients were 100% (23/23). Therefore, gastrointestinal endoscopists are required to have relatively skilled operation experience.

The early complications of stent implantation are mainly bleeding, perforation, stent displacement or slippage, and abdominal pain caused by intestinal wall injury, among which intestinal wall perforation is the most serious, which is easy to cause severe abdominal infection and secondary septic shock, so emergency surgery is required [26]. This study had no apparent complications except for two patients with rectosigmoid junction cancer who had stent migration. In the two patients, the symptoms of intestinal obstruction were gradually relieved during the transitional period before the migration of the inserted stent, and the subsequent laparoscopic surgery was successfully performed. Laparoscopic colorectal cancer surgery has the advantages of less trauma and rapid postoperative recovery and has gradually become the standard mainstream operation for colorectal cancer treatment. Corresponding randomized controlled studies worldwide have shown that compared with open surgery for colon cancer. Laparoscopic surgery has obvious short-term efficacy advantages, such as less intraoperative blood loss, less postoperative pain, faster recovery of intestinal function, and shorter hospital stay [27].

Moreover, the long-term follow-up results of these studies found no significant difference in the 3-year disease-free survival rate and 3-year overall survival rate between laparoscopic and open colon cancer surgery, and the stratified analysis according to tumor stage did not reflect the difference between the two [28]. Stent implantation does not affect the subsequent laparoscopic operation and the survival rate of patients, so laparoscopic surgery after stent implantation has good safety and short-term efficacy in treating left-sided colon cancer obstruction [29]. Of the 23 patients in the experimental group, 22 patients successfully underwent laparoscopic left hemicolectomy and primary anastomosis, and only one 81-year-old female patient underwent laparoscopic radical resection of sigmoid colon cancer + distal closure + proximal ostomy due to poor bowel preparation and general condition deviation.

This study also showed no significant differences in operation time, intraoperative blood loss, number of harvested lymph nodes, and postoperative anal exhaust time between the two groups (all *P* > 0.05). However, the overall postoperative complication rate and hospital stay in the metal stent implantation combined with the laparoscopic surgery group were significantly lower than those in the Hartmann’s surgery group (*P* < 0.05). Further subgroup analysis of the overall incidence of postoperative complications in the two groups showed that the traditional Hartmann’s procedure group was likelier to have an incomplete intestinal obstruction (*P* < 0.05). It was considered that although the intestinal tumor had been removed and the complete obstruction had been relieved after the emergency Hartmann’s operation, the large range of intestinal dilatation and inflammatory edema in the state of intestinal obstruction still needed to continue for some time to disappear completely. So our research results also verify the metal stent implantation under endoscopy combined with X-ray fluoroscopy joint the feasibility of laparoscopic surgery in treating acute left half colon cancer obstruction, compared with the traditional emergency laparotomy, which is more minimally invasive, safe, and effective. Moreover, this combined technology can avoid the traditional second or even third-surgical trauma and improve patients’ quality of life so that patients can recover faster after surgery, to benefit patients.

This study also found no significant differences in overall survival and recurrence-free survival between the observation and control groups (all *P* > 0.05). Therefore, there is no significant difference in the long-term prognosis between the endoscopic and X-ray-guided metal stent placement combined with laparoscopic surgery for acute left colon cancer obstruction and the traditional Hartmann surgery group. In a study conducted by Takahashi et al., it was found that there is no significant variance in long-term survival rates between patients who underwent stenting and those who chose surgical intervention for palliation in cases of obstructing unresectable colon cancer. Interestingly, stenting seemed to correlate with more favorable patient prognoses [30]. Similarly, the work of Ueki et al. indirectly implied that the overall survival rates and recurrence-free survival rates were similar between patients who had stent placement and those who did not [31]. Building upon these findings, our research, in conjunction with the study conducted by Verstockt et al., further supports the notion that utilizing stenting as a bridge to surgery for obstructing colorectal cancer does not lead to inferior survival outcomes for patients treated with curative intent [32]. These results highlight the positive impact of stenting as a viable treatment option for patients with obstructing colorectal cancer. Because our sample size is small, more studies with larger sample sizes will be needed to ascertain our results further.

## Conclusions

The comparison of the therapeutic effects of the two groups verified the feasibility of endoscopic combined with X-ray fluoroscopy metal stent placement in combination with laparoscopic surgery in the treatment of acute left-sided colon cancer obstruction. Compared with the traditional emergency open Hartmann’s procedure, metal stent implantation under endoscopy combined with X-ray fluoroscopy combined with laparoscopic surgery is more minimally invasive, safe, and effective and avoids the traditional second or even third surgical trauma to effectively improve the quality of life of patients, so that patients can recover quickly after surgery.

## Data Availability

All data generated or analyzed during this study are included in this published article. Further information can be obtained from the corresponding author on reasonable request.

## Abbreviations

BMI: Body mass index
TNM: Tumor–node–metastasis
OS: Overall survival
RFS: Recurrence-free survival
